# Plant Stress Responses and Phenotypic Plasticity in the Epigenomics Era: Perspectives on the Grapevine Scenario, a Model for Perennial Crop Plants

**DOI:** 10.3389/fpls.2017.00082

**Published:** 2017-02-06

**Authors:** Ana M. Fortes, Philippe Gallusci

**Affiliations:** ^1^Faculdade de Ciências, Instituto de Biossistemas e Ciências Integrativas, Universidade de LisboaLisboa, Portugal; ^2^UMR EGFV, Université de Bordeaux, Institut national de la recherche agronomique, Institut des Sciences de la Vigne et du VinVillenave-d’Ornon, France

**Keywords:** DNA methylation, epigenomics, grape, Histone Post-Translational Modifications, small RNAs, *Vitis vinifera*

## Abstract

Epigenetic marks include Histone Post-Translational Modifications and DNA methylation which are known to participate in the programming of gene expression in plants and animals. These epigenetic marks may be subjected to dynamic changes in response to endogenous and/or external stimuli and can have an impact on phenotypic plasticity. Studying how plant genomes can be epigenetically shaped under stressed conditions has become an essential issue in order to better understand the molecular mechanisms underlying plant stress responses and enabling epigenetic in addition to genetic factors to be considered when breeding crop plants. In this perspective, we discuss the contribution of epigenetic mechanisms to our understanding of plant responses to biotic and abiotic stresses. This regulation of gene expression in response to environment raises important biological questions for perennial species such as grapevine which is asexually propagated and grown worldwide in contrasting *terroirs* and environmental conditions. However, most species used for epigenomic studies are annual herbaceous plants, and epigenome dynamics has been poorly investigated in perennial woody plants, including grapevine. In this context, we propose grape as an essential model for epigenetic and epigenomic studies in perennial woody plants of agricultural importance.

## Introduction

Epigenetic mechanisms regulate chromatin structure, gene expression, transposon mobility and DNA recombination (He et al., 2011; Pikaard and Scheid, 2015). They generally refer to modifications of gene expression that can be inherited through mitosis or meiosis yet without changes in the underlying DNA sequences (Eichten et al., 2014) and also include chromatin modifications that may lead to stable alteration of the transcriptional programming of non-dividing cells even after removal of the triggering signals (Avramova, 2015).

Epigenetic regulation is mediated by a complex interplay among different molecular actors. These include the DNA methylation/demethylation machinery, enzymes mediating histone post-translational modifications (PTMs), the remodeling of chromatin organization and specific classes of small RNAs and long non-coding RNAs (Lauria and Rossi, 2011; Pikaard and Scheid, 2015; Gallusci et al., 2016). Briefly, in plants 5 methyl-cytosine (m^5^C) is found in all sequence context, including the CG and CHG (H = A, T, or C) symmetrical motives and the non-symmetrical CHH motif (reviewed in Gehring, 2013). DNA methylation is maintained in a post-replicative way by three classes of DNA methyltransferases: DNA METHYLTRANSFERASE 1 (MET1) and CHROMOMETHYLASE 3 (CMT3) for CG and CHG contexts, respectively, and by the DOMAIN REARRANGED METHYLTRANSFERASE 2 (DRM2), which requires an siRNA guide and reestablishment after each cycle of DNA replication or by CMT2 for the asymmetric CHH context (Du et al., 2012; Matzke and Mosher, 2014). Finally, DNA methylation can be lost after replication when maintenance of DNA methylation is not functional or actively reversed by DNA Glycosylase-Lyases (Piccolo and Fisher, 2014).

Histone PTMs are also essential epigenetic signals that can occur at the N-terminal tail of core histones (H2A, H2B, H3, H4) through acetylation, methylation, phosphorylation and ubiquitination (Berr et al., 2011). Histone acetylation and methylation at lysine residues are established by histone acetyltransferases (HATs) and histone lysine methyltransferases (HKMTs), respectively, which are encoded by complex multigenic families. These epigenetic marks can be removed by histone deacetylases (HDACs) and histone demethylases (HDMs), respectively (Berr et al., 2011; He et al., 2011; Pikaard and Scheid, 2015).

The recent development of epigenome profiling has boosted our understanding of the dynamics and function of epigenetic marks in plants. Several approaches have been recently developed (Schmitz and Zhang, 2011; Lee and Kim, 2014^1^). So far, histone PTM analysis relies on Chromatin Immunoprecipitation (ChIP) using specific antibodies followed by hybridization to tilling arrays (ChIP- chip, Makarevitch et al., 2015) or by high throughput sequencing (ChIP-Seq, Wang et al., 2009). DNA methylation landscape can be studied by making use of methyl sensitive restriction enzyme to enrich DNA in methylated or un-methylated sequences that are subsequently hybridized to tilling arrays or sequenced (Kim et al., 2014). Alternatively, methylated regions can be selected using m^5^C specific antibodies (MeDIP), and analyzed with tilling arrays (MeDip-ChIP) or by Next Generation Sequencing (Medip Seq). Both approaches were used for methylome analysis for example in *Arabidopsis*, or poplar (Zhang et al., 2006; Zilberman et al., 2006; Kim et al., 2014). In particular, Medip-Seq was used to analyze the changes in methylation patterns during *in vitro* culture of cassava (Kitimu et al., 2015). But the golden standard for methylome analysis is the combination of bisulfite conversion of DNA to high throughput sequencing that allows analyzing the methylation landscape at a single base resolution (Whole Genome Bisulfite sequencing: WGBS). The methylomes of *Arabidopsis* (Cokus et al., 2008; Lister et al., 2008; Stroud et al., 2013), rice (Li et al., 2012; Garg et al., 2015), maize (Eichten et al., 2013), tomato (Zhong et al., 2013), *Brassica* (Chalhoub et al., 2014) and many others (Niederhuth et al., 2016) have now been described using this approach.

In this perspective, we will firstly focus on the analysis of the genome wide distribution of epigenetic marks in plants under stresses. However, most species used for epigenomic studies are annual herbaceous plants and little is known about epigenomes in perennial woody plants. Indeed, omics’ approaches have been initiated in grape to understand environmental effects on plant and fruit development (Fortes et al., 2011; Agudelo-Romero et al., 2015). In addition, a few studies have indicated that epigenetic mechanisms might be involved in various aspects of grape development (Aquea et al., 2011). However, knowledge of grape epigenomes and of their variation has remained very limited until now (Niederhuth et al., 2016). Yet, grapevine presents several features that make it a relevant model for the study of epigenetic mechanisms due to the fact that is a perennial woody plant and the fruit maturation is subjected to non-climacteric molecular and hormonal regulation (Fortes et al., 2015). Grapevine varieties are preserved in their distinct genetic backgrounds through clonal propagation. However, phenotypic diversity exists within clones (Pelsy, 2010) that is unlikely to be solely driven by differences in DNA sequence. These facts contribute to the relevance of grape as a model for epigenetic and epigenomic studies in perennial woody plants of agricultural importance.

## Epigenetic Reprogramming During Abiotic Stress Responses

Recent studies have shown the differential regulation of genes encoding epigenetic regulators (Fang et al., 2014; Li et al., 2014; Su et al., 2015) as well as local chromatin and DNA methylation changes in response to a variety of abiotic stresses including cold, salinity, drought, osmolality, or mineral nutrition, thereby highlighting the relevance of epigenetic regulations in these contexts (Chen et al., 2010; Luo et al., 2012; González et al., 2013; Bocchini et al., 2015; Kim et al., 2015; Liu et al., 2015). Consistent with these results, genome wide analyses of histone PTMs and DNA methylation distribution have revealed global epigenomic reprogramming in plants under abiotic stresses. In a recent study, trimethylation at lysine 4 on histone 3 (H3K4me3), a mark normally associated with gene expression, was analyzed in *Arabidopsis* plants under drought stress using ChIP-seq and showed to be highly dynamic and positively correlated with the transcription level of drought induced genes in response to stress (Dijk et al., 2010). Similar results were found in rice (Zong et al., 2013) and in moss (Widiez et al., 2014). Osmotic stress also causes an increase in phosphorylated histone H3 threonine 3 (H3T3ph) located at pericentromeric regions where it is thought to help maintaining the heterochromatin structure (Wang et al., 2015). Interestingly, H3T3ph is also present in active genes where it seemed to antagonize H3K4me3, suggesting that H3T3ph may have a repressive function on gene expression during osmotic stress (Wang et al., 2015) a role also suggested for histone deacetylase HDA9 (Zheng et al., 2016). In addition, priming effects in *Arabidopsis* were shown to be partly mediated by remodeling of the epigenomic landscape, and involves the repressive mark H3K27me3 (Sani et al., 2013).

Recently, a specialized histone H1 variant was shown to be required for a substantial part of DNA methylation associated with environmental stress in *Arabidopsis* (Rutowicz et al., 2015) and two DEAD-box RNA helicases were suggested to be involved in epigenetic silencing of gene expression leading to suppression of *Arabidopsis* stress response (Khan et al., 2014).

In addition, DNA methylation is also critical for the responses of plant to abiotic stresses. This was initially shown by the demonstration that *Arabidopsis* mutants deficient in various steps of the RdDM pathway or in CHG maintenance methylation are affected in their capacity to modulate the stomatal index under low relative humidity (Tricker et al., 2012), present an hypersensitivity to heat exposure (Popova et al., 2013) or an enhanced sensitivity to phosphate starvation (Yong-Villalobos et al., 2015). These results are consistent with an important function of the DNA methylation dynamics in the regulation of abiotic stress–responsive genes. Indeed drought stress, but also nutrient deprivation cause extensive remodeling of DNA methylation patterns in *Arabidopsis* (Colaneri and Jones, 2013; Yong-Villalobos et al., 2015; Wibowo et al., 2016), barley (Chwialkowska et al., 2016) or *Populus* (Liang et al., 2014). In this latter case, modulation of DNA methylation at repetitive elements appeared essential for the control of adjacent gene expression (Liang et al., 2014) a function also suggested in maize where TEs could be used as local enhancers for stress responsive genes (Makarevitch et al., 2015). Similarly Pi deficiency in rice modulates DNA methylation at TEs located close to genes highly induced under this stress (Secco et al., 2015). In this case, however, TEs were hyper-methylated an event that occurred after gene induction most likely to prevent potentially deleterious activity of TEs located in the vicinity of highly induced stress responsive genes.

As a conclusion, the results discussed above are consistent with the idea that abiotic stresses cause significant reprogramming of chromatin not only related to gene expression, but also to the control of chromosome organization. In addition, evidence of transgenerational inheritance of plant responses to stress has been provided (Tricker et al., 2013; Migicovsky et al., 2014); although this process appears limited to and mainly mediated by the female gamete (Wibowo et al., 2016).

## Epigenetic Reprogramming During Plant Biotic Stress Responses

Regarding histone Post-Translational Modifications and DNA methylation occurring upon biotic stress there is lesser information available than for abiotic stress. However, recent findings indicate that chromatin modifications contribute to plant immunity against both necrotrophic and biotrophic pathogens (reviewed by Ding and Wang, 2015). In fact, the expression of R genes which are central regulators of plant immunity was shown to be regulated by *Arabidopsis* E3 ubiquitin ligase genes *HISTONE MONOUBIQUITINATION1 (HUB1)* and *HUB2* (Zou et al., 2014). Histone monoubiquitination at the R gene locus had an impact on immune responses. The loss of- function mutant *bon1-1* has enhanced disease resistance to the virulent pathogen Pst DC3000 and both *HUB1* and *HUB2* mediate its autoimmune responses. In another case, HDA19, an *Arabidopsis* histone deacetylase, was shown to play a negative role in basal defense mediated by the SA-dependent signaling pathway. Loss of *HDA19* causes increased expression of SA biosynthetic genes and defense genes and promotes resistance to the virulent Pst DC3000 (Choi et al., 2012). Dimethylated or trimethylated histone H3 Lys 27 (H3K27me2/3) marks silent or repressed genes involved in stress responses in plants. Li et al. (2013) showed that the rice Jumonji C protein gene JMJ705 encodes a histone Lys demethylase that specifically reverses this mark. An increase in JMJ705 expression in transgenic plants removes H3K27me3 from defense-related genes, induces their expression with involvement of jasmonic acid, and enhances plant resistance to biotic stress. Interestingly, Soyer et al. (2014) showed that chromatin-based transcriptional regulation can also act on effector gene expression in fungi during plant infection. Pathogen infection has been also reported to change histone modifications in some defense response genes (De-La-Peńa et al., 2012).

The profiling of the DNA methylomes of plants exposed to bacterial pathogen, avirulent bacteria, or salicylic acid revealed numerous stress-induced differentially methylated regions (DMRs) often coupled to differential gene expression (Dowen et al., 2012). Mutant plants globally defective in maintenance of CG methylation (*met1-3*) or non-CG methylation (*ddc*, *drm1-2 drm2-2 cmt3-11*) were markedly resistant to bacterial colonization.

DNA demethylation likely primes transposable elements as well as defense gene induction through the concomitant activation of their transactivators and/or the interference with other chromatin marks (Yu et al., 2013). Some immune-response genes, containing repeats in their promoter regions, are negatively regulated by DNA methylation. These defense gene loci may lose DNA methylation so that they are more easily activated at the transcriptional level (Yu et al., 2013). This is corroborated by the study of Le et al. (2014); the DNA methylases ROS1, DML2, and DML3 were shown to play a role in fungal disease resistance in *Arabidopsis* since a triple mutant *rdd* (*ros1 dml2 dml3*), presents down-regulation of stress response genes and increased susceptibility to a fungal pathogen. Furthermore, these authors showed that DNA demethylases target promoter transposable elements in stress responsive genes to positively regulate them.

## Natural and Induced Epigenomic Variation, Phenotypic Plasticity and Breeding

**FIGURE 1:**
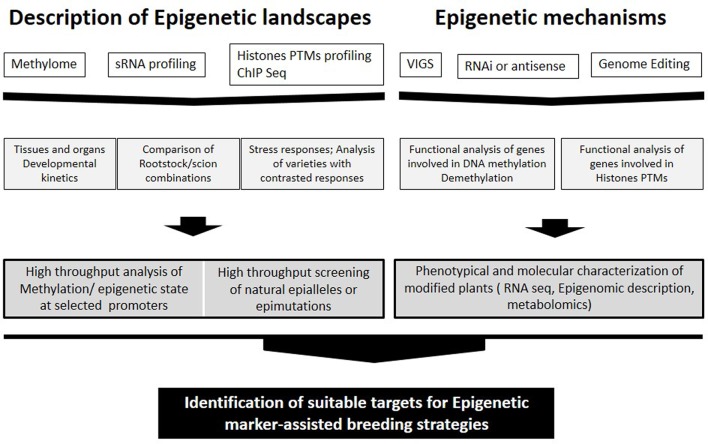
**Approaches for identification of suitable targets for Epigenetic marker-assisted breeding strategies ranging from studying epigenetic landscapes to clarification of epigenetic mechanisms**.

Natural epigenomic variation occurs during species evolution (Hirsch et al., 2013) and together with genetic variation is likely involved in the phenotypic diversity and plasticity of plants. Epigenetic variation is sensitive to environmental inputs; epialleles induced by the environment or experimentally may be formed at a higher rate than alleles generated from genetic variation and may also be inherited leading to better adaptation to the environment (**Figure 1**; Hirsch et al., 2013).

Experimentally induced epialleles have been produced in *Arabidopsis* by generating Epigenetic Recombinant Inbred Line (EpiRILs) populations derived from *decrease in DNA methylation 1-2* (*ddm1-2*) or the *met1* parents (Johannes et al., 2009; Reinders et al., 2009). EpiRILs were subsequently used to identify epiQTL corresponding to DMRs that determine two complex traits, flowering time and primary root length (Cortijo et al., 2014). Interestingly, these EpiRILs present variation in growth capacity (Hu et al., 2015) and are more sensitive to salinity stress than the Col0 parent line suggesting that *ddm1* derived epigenotypes limit the ability to adapt to this stress (Kooke et al., 2015). As an alternative approach, a stochastically hypomethylated population was generated by selfing *Brassica rapa* plants previously treated with the demethylating agent 5-Azacytidine (Amoah et al., 2012). This population was used for forward screening of agronomic traits such as flowering time, seed protein content and fatty acid components. These results suggest that a portion of QTLs that have been used by breeders so far may be due to epigenetic, rather than genetic variation (Springer, 2013).

DNA methylation may also have an important role in the long term adaptation of plants (**Figure 1**; Garg et al., 2015). Two rice cultivars with contrasting sensitivity to drought stress and salinity showed clearly different methylation landscapes; part of the DMRs between cultivars were associated with genes involved in stress responses (Garg et al., 2015).

Indeed variation in methylation patterns have been also observed in natural populations and might be associated with specific environmental traits. In a recent study, Dubin et al. (2015) showed by analyzing *Arabidopsis* accessions from Northern and Southern Sweden that CHH methylation at transposons increases with temperature and this was associated with major genetic variants at the CMT2 locus. In the same study, Gene Body Methylation which was not modified by temperature was shown to be correlated with the latitude of origin; Southern accessions being less methylated than Northern one. This was associated with a lower expression of the targeted genes in Southern accessions consistent with local adaptation of the accessions.

Epialleles impacting plant traits have now been identified in many plants (Rodríguez López and Wilkinson, 2015) since the initial characterization of the *cycloidea* and *Cnr* epimutations in snapdragon and tomato, respectively (Cubas et al., 1999; Manning et al., 2006; Poole et al., 2006). For example, Vitamin E in tomato is determined by epigenetic variations linked to a SINE retrotransposon located in the promoter region of a gene involved in the vitamin synthesis. This work showed that naturally occurring epialleles may be responsible for regulation of nutritionally important metabolic QTLs and determination of agronomic traits (Quadrana et al., 2014). In another study, the complex trait of Energy use efficiency was shown to possess an epigenetic component that is stably inherited, allowing the creation of distinct isogenic sublines that can be used in breeding (Hauben et al., 2009). Thus, induced or natural epigenetic diversity may represent an unexplored resource of phenotypical variations that could be used in plant breeding programs, as recently discussed in Rodríguez López and Wilkinson (2015).

## Grapevine Epigenomics and Epigenetics: A Model Plant for Perennial Crop Plant

Studies on *Arabidopsis* revealed functional aspects of epigenetic regulation of gene expression but present limitations since *Arabidopsis* has only 5% of methylated cytosine in the genome whereas many crops contain more than 20% (Lee and Kim, 2014). In fact, mutations in epigenetic regulators seem to have a higher impact in crops than in *Arabidopsis* (Mirouze and Vitte, 2014; Gallusci et al., 2016). In addition, *Arabidopsis* contains very few transposable elements comparing to crops (reviewed by Lee and Kim, 2014). Polymorphisms in transposon insertions and repeats can originate natural epigenetic variation. Furthermore, while the distribution of the genes along the chromosomes of *Arabidopsis* is fairly homogeneous, this situation may differ in crops. For example, *Vitis vinifera* genome is characterized by alternation of large regions with high and low gene density (Jaillon et al., 2007).

Several studies have already emerged in crops, in particular, recent analyzes carried out in tomato fruits (Zhong et al., 2013; Liu et al., 2015) constitute a relevant background for studies in grape. It is not yet known whether the epigenetic control of ripening is similar in all fleshy fruits or is limited to the tomato and related wild species (reviewed in Gallusci et al., 2016). Nevertheless, the expression patterns of several genes involved in DNA methylation and histones modifications indicate that epigenetic factors are involved in the onset of *véraison* in grape and a global decrease in DNA methylation may eventually occur during grape ripening (Fortes et al., 2011) as reported for tomato (Zhong et al., 2013; Liu et al., 2015). In this context, the lack of available mutants in grape constitutes a limitation comparing to tomato. However, studies addressing the methylation status of promoters of genes involved in easily identified traits can shed light on epigenetic regulation of gene expression in grape (**Figure 1**).

Chemical treatments that affect DNA methylation patterns could also be utilized to generate epimutations (Amoah et al., 2012) though they may not be as stable as genetic mutants. Several examples of epimutations in crops are mentioned in the review by Zhang and Hsieh (2013). Epimutagenesis may allow the opportunity to explore allelic variation and novel combinations of alleles without relying upon recombination (Springer, 2013).

Analysis of the distribution of epi-marks and DNA methylation in grape in relation with gene expression profiles and fruit quality traits would likely identify epialleles that could be used as important new targets for plant breeding (**Figure 1**). DNA methylation may generate multiple epialleles with various expression levels, thereby leading to continuous quantitative variation of a trait (Zeng and Cheng, 2014). On the other hand, Kitimu et al. (2015) identified candidate epimarks that distinguish between field cuttings and meristem culture cassava samples. Specific methylation signatures may be used in the future for the diagnosis of somaclonal variants and clonal stocks in grapevine.

Grape combines several specific features that could make it an appealing model to study epigenetic regulations in woody perennial plants. It is used as one of the main models for non-climacteric fruits and also flower development is programmed 1 year in advance; the impact of environmental conditions on flower and subsequently fruit development seems to be in part determined by the environmental conditions the year before. Grape also has specific requirements such as grafting, and clonal propagation. In this context, epigenetic variability could add to the genetic diversity of grape to shape the phenotypic variations observed in this plant. Consistent with this view, clonal diversity within *V. vinifera* varieties has been distinguished using the methylation-sensitive amplified polymorphism technique, highlighting the usefulness of using epigenetic markers in intra-varietal diversity studies (Ocaña et al., 2013). Grafting could also impact the epigenetic state of both rootstocks and shoots (scions), **Figure 1**. Recently, Lewsey et al. (2015) showed that mobile sRNAs regulate the DNA methylation landscape genome wide, and may be an important mechanism of genome defense in crops. They showed that site-specific transmission of epiallelic states from one accession to another can be achieved by grafting and by *de novo* methylation of unmethylated DNA, consistent with the idea that some effects of grafting are due to the movement of small RNAs. In grapevine, grafting with rootstocks induced the up-regulation of genes associated with DNA methylation and chromatin modification in the shoot apical meristem (Cookson and Ollat, 2013). Clarifying these mechanisms may open doors to innovative applications to enhance grapevine tolerance to stresses and grape quality.

In line with these ideas, the recent analysis of the transcriptomic changes associated with grape infection with the necrotrophic pathogen *Botrytis cinerea* suggested that epigenetic mechanisms are involved in the reprogramming of fruit defense (Agudelo-Romero et al., 2015). Genes coding for histones, DNA (cytosine-5)-methyltransferase, helicases, DICER and ARGONAUTE proteins were modulated during the infection, whereas those associated with TEs mobility were down-regulated (**Table 1**).

**Table 1:** Genes involved in epigenetic mechanisms differentially modulated in Trincadeira grapes infected with the fungus *Botrytis cinerea* at green hard stage (EL33) and *véraison* stage (EL35).

12X V1 ID	EL33Inf/EL33Mock significant Fold Change	EL35Inf/EL35Mock significant Fold Change	Functional annotation
VIT_13s0064g01340	**3,1**	**2,4**	Histone H3
VIT_07s0005g01060		**3,0**	Histone H1
VIT_06s0004g03890		**2,2**	Histone H4
VIT_04s0023g03130		**2,2**	Histone H1
VIT_08s0007g00040		**2,2**	Histone H4
VIT_12s0035g00060		**-3,8**	DNA (cytosine-5)-methyltransferase
VIT_12s0034g02560		**-4,0**	DNA (cytosine-5)-methyltransferase (ATHIM)
VIT_06s0004g02600	**-2,3**		MOM1 (maintenance of methylation1)
VIT_01s0010g00020	**-2,5**	**2,0**	DNA-3-methyladenine glycosidase I
VIT_17s0000g04900	**-2,2**		ATP-dependent RNA helicase
VIT_05s0020g03760	**-2,2**		RNA helicase SDE3 (SDE3)
VIT_01s0010g00690	**-2,2**		DNA-directed RNA polymerase
VIT_11s0016g03220	**-3,9**		RNA-directed RNA polymerase
VIT_14s0006g00760		**2,2**	ATP-dependent RNA helicase
VIT_01s0010g03200	**-2,2**		DNA-directed RNA polymerase (RPOT2)
VIT_00s0794g00010	**-2,1**		DEAD/DEAH box RNA helicase protein RH16
VIT_15s0048g02380	**-2,1**		DCL1 (DICER 1)
VIT_10s0042g01150	**-2,6**		ARGONAUTE 2 (AGO2)
VIT_02s0025g03560	**-2,8**		Transcription factor jumonji (jmjC) DIDO1
VIT_02s0012g01960		**2,8**	Transcription factor jumonji (jmj)
VIT_11s0149g00100	**-2,3**		DICER-like 4
VIT_04s0008g06930	**2,2**		Transposase, IS4
VIT_14s0036g01410	**-2,1**		Gag-pol polyprotein
VIT_03s0038g02730	**-2,3**		Mutator-like transposase
VIT_07s0130g00290	**-2,5**		Transposase, IS4
VIT_04s0069g00030	**-2,9**		Retrotransposon protein
VIT_00s0227g00030	**-3,0**		Gag-pol polyprotein

*Details on microarray analysis available in Agudelo-Romero et al. (2015)*.

Base-resolution methylomes and high-throughput sRNA profilings are already available in more than 34 species (Niederhuth et al., 2016) including *V. vinifera*. Comparing the epigenomes of wild and cultivated *Vitis* species with and without biotic and non-biotic stresses will bring insights on the epigenetic basis of grapevine resistance to adverse conditions with potential impact in breeding strategies. Moreover, epigenetic marks may participate in the priming mechanisms to better withstand biotic and abiotic stresses (Crisp et al., 2016), another topic that deserves attention in order to moderate stress susceptibility and increase climate change resilience in grapevine. Interestingly, these epimarks can also be used in the future for distinguishing agronomic practices and *terroir* certification of wines.

Previously, transgenerational systemic acquired resistance, was demonstrated to be a prominent defense mechanism toward downy mildew pathogen and involves DNA methylation (Luna and Ton, 2012). In grapevine, a further layer of complexity can be added since memory in perennial plants is affected every year in meristems committed to flowering. Furthermore, the reason why epigenetic regulation in response to stress can be transient or transgenerational are not clear (Tricker, 2015). It is also not known the contribution of pathogen-responsive siRNAs in transgenerational immune priming and how they drive the selection of new phenotypes especially in perennial plants.

A deeper understanding of the molecular mechanisms involving tissue-specific epigenetic changes underlying genotype × environment interactions may be beneficial for long-term improvement of grapevine performance in less predictable climates with new sources of diseases.

In a near future, epigenetic marker-assisted breeding strategies will be applied to select for agronomical desirable epigenetic quantitative traits (**Figure 1**). Crop improvement via locus-specific epigenetic manipulation has become increasingly feasible with TALE- or CRISPR-based genome editing technologies (Mendenhall et al., 2013; Zhang and Hsieh, 2013). Such technologies can be expected to play an important role in grapevine improvement once transgenesis’ protocols are optimized for different cultivars.

## Author Contributions

AF and PG designed the perspective and wrote the manuscript.

## Conflict of Interest Statement

The authors declare that the research was conducted in the absence of any commercial or financial relationships that could be construed as a potential conflict of interest.

## Footnotes

1 https://www.plant-epigenome.org/
